# Effectiveness of a gentamicin impregnated collagen sponge on reducing sternal wound infections following cardiac surgery: a meta-analysis of randomised controlled trials

**DOI:** 10.1308/003588412X13171221590179

**Published:** 2012-05

**Authors:** S Creanor, A Barton, A Marchbank

**Affiliations:** ^1^Plymouth UniversityUK; ^2^Research Design Service – South WestUK; ^3^Plymouth Hospitals NHS TrustUK

**Keywords:** Cardiac surgery, Sternal wound infections, Gentamicin impregnated collagen sponge

## Abstract

**INTRODUCTION:**

Gentamicin impregnated collagen sponges are licensed for use after cardiac surgery in over 50 countries but their effectiveness at preventing sternal wound infections (SWIs) remains uncertain. The aim of this meta-analysis was to assess the current evidence for effectiveness of such sponges at preventing SWIs in patients after cardiac surgery.

**METHODS:**

A systematic search of the literature was undertaken and meta-analyses were performed on the results of the identified, eligible studies. Using random effects models, odds ratios (OR) and corresponding 95% confidence intervals (Cl) were calculated for all SWIs and deep SWIs for: a) all participants, and b) participants deemed as high risk.

**RESULTS:**

Three unique randomised controlled trials (published between 2005 and 2010) involving 3,994 participants met the inclusion criteria. There was insufficient evidence of a significant difference between intervention and control groups for all SWIs (all participants: OR: 0.66, 95% Cl: 0.39–1.14; high risk participants: OR: 0.60, 95% Cl: 0.24–1.52). There was insufficient evidence of a significant benefit of the sponge in deep SWIs across all participants (OR: 0.72, 95% Cl: 0.47–1.10) but some evidence of benefit in terms of reducing the incidence of deep SWIs in high risk participants (OR: 0.62, 95% Cl: 0.39–0.98).

**CONCLUSIONS:**

There is insufficient evidence of the effectiveness (or otherwise) of gentamicin impregnated sponges in preventing SWIs following cardiac surgery. However, some evidence does exist that such sponges can reduce the incidence of deep infections in high risk patients.

More than 55,000 cardiac operations through median sternotomy are performed every year in the UR,^1^ aiming to improve survival and quality of life. Despite the now routine use of prophylactic systemic antibiotics, sternal wound infection (SWI) is a complication affecting between 0.9% and 20% of patients after cardiac surgery as reported in various series.^2^ SWI may affect outcome adversely and result in serious consequences for both the patient and the healthcare system. It is estimated that 500–600 patients per year will require reoperations for deep SWI and over 40 of these patients will die.^1^ SWI leads to prolongation of hospital stay, the need for treatment with antibiotics and vacuum dressings, and increased hospital costs and occupation of hospital beds.

SWIs can be categorised as either superficial or deep. Superficial infection affects only the presternal tissues while deep infection includes sternal osteomyelitis and mediastinitis. Deep SWI is a devastating complication. A study from one major UR centre reported that 1.65% of the patients who underwent cardiac surgery between 2001 and 2005 required reoperations for deep SWI and 9.2% of them died.^3^ The median hospital stay in these patients was 52 days, compared with only 8 days for patients without a deep infection.

The incidence of superficial wound infections may be twice that of the deep infections, with median and mean hospital stays of 12 and 15 days respectively.^2^^–^^4^ From the previously published data referred to above, overall, it could be estimated that 1,500–2,000 patients every year will suffer an SWI after cardiac surgery in the UR. Apart from the associated patient morbidity and mortality, there is also significant financial cost. Every extra bed day costs a minimum of £280, which increases to over £1,000 for an intensive care unit bed. There is therefore a continued need for investigating new methods to prevent as well as treat SWIs.

Gentamicin impregnated collagen sponges have been developed to prevent and treat wound infections and the sponges are currently licensed for use after cardiac surgery in over 50 countries. There is a paucity of evidence to suggest their routine use is justified in the UR and, in common with other interventions, it may be that the upfront cost of the implant constitutes a barrier to their widespread implementation. The aim of this meta-analysis was to assess the current evidence for effectiveness of gentamicin impregnated collagen sponges at preventing SWIs in patients after cardiac surgery.

## Methods

A quantitative review of the literature was undertaken to identify relevant prospective randomised trials. The following databases were searched from inception to June 2011: the British Nursing Index, PubMed Central®, CINAHL® (Cumulative Index to Nursing and Allied Health Literature), Embase™ and MEDLINE®. The search strategy was kept as wide as possible and consisted of a combination of the following free text words and MeSH (Medical Subject Headings) terms (with synonyms and closely related words): thoracic surgery, heart surgery, cardiovascular surgery, collagen, surgical sponges, absorbable implants, wound infection. There were no restrictions concerning the language of the article or publication type. Reference lists of the retrieved articles were checked for further publications.

Studies were included in the meta-analysis if they were randomised controlled trials (RCTs) investigating the effectiveness of antibiotic sponges compared with a placebo or no intervention in patients undergoing cardiac surgery. The data of all participants, irrespective of type of cardiac sur gery and length of follow-up period, were considered. Two authors scanned the titles and abstracts of the articles retrieved by the initial screening search to exclude obviously ineligible studies (eg non-randomised studies). The full text articles of the remaining studies were read by two authors who decided independently whether these studies met the inclusion criteria. AH studies deemed to meet the inclusion criteria were included in the meta-analysis.

Together with details such as title, authors, country of study, length of follow-up period of each study, all outcome data related to post-operative SWIs were extracted. In order to include as many studies as possible, ‘any SWI’ was defined as the primary outcome for this meta-analysis. ‘Deep SWI’ was considered separately as a secondary outcome. As well as considering all participants, meta-analyses were also undertaken on the subgroup of participants defined as being ‘high risk’. The outcome data were entered into Rev-Man version 5.1.0 (Nordic Cochrane Centre, Copenhagen, Denmark) for the pooled statistical analyses.

Both fixed and random effects models were examined, with random effects models being presented. For each model, the odds ratio (OR) and corresponding 95% confidence interval (Cl) were calculated. Evidence of a significant effect of the intervention (ie gentamicin sponge) was concluded if the Cl did not include the value 1. Statistical heterogeneity was assessed with the I^2^ statistic and assumed to exist if I^2^>40%.^5^ Different analyses were planned *a priori*to explore relevant subgroups (all SWI and deep SWI; all participants and high risk participants).

## Results

Three randomised trials were identified that fulfilled the inclusion criteria.^6^^–^^8^ Rey characteristics of these trials are given in Table 1. Two studies were in Scandinavia^6^^–^^7^ and one was in the US.^8^ Two studies had a follow-up period of three months^6^^–^^8^ while one study had a shorter follow-up period of two months.^7^ In each trial, the aim was to establish the effectiveness of a gentamicin impregnated collagen sponge, in addition to systemic antibiotics, in preventing post-operative SWIs. The primary outcome was defined similarly in each trial, based around the 1992 SWI definition of the US Centers for Disease Control and Prevention.^9^

**Table 1:** Main characteristics of included studies assessing the effectiveness of gentamicin impregnated collagen sponge in reducing post-operative sternal wound infection

Reference	Patient group	Number of participants	Number of centres	Gentamicin sponge dose	Patient blinded?	Assessor blinded?	Definition of SWI	Follow-up duration
Eklund *et al*^6^	Elective CABG	542	1 (Finland)	130mg gentamicin (1 sponge)	No	Yes	CDC 1992	3 months
Friberg *et al*^7^	Cardiac surgery through median sternotomy	1,950	2 (Sweden)	260mg gentamicin (2 sponges)	Yes	Yes	CDC 1992 (minor modifications)	2 months
	High risk subgroup: DM and/or BMI>25kg/m^2^	1,385						
Bennett-Guerrero *et al*^8^	Elective CABG +/- AVR *and* high risk: DM and/ or BMI >30kg/m^2^	1,502	48 (US)	260mg gentamicin (2 sponges)	Yes	Yes (consensus of 3 blinded assessors)	CDC 1992 + asepsis	90 days

SWI = sternal wound infection; CABG = coronary artery bypass graft; CDC = Centers for Disease Control; DM = diabetes meli itus; BMI = body mass index; AVR = aortic valve replacement

From these three RCTs, a total of 3,994 participants undergoing cardiac surgery had been randomly allocated to intervention (ie receiving a gentamicin impregnated sponge prior to the closure of the sternum) or control groups. The number of participants in each trial ranged from 270 to 983 per group. Two of the studies included participants defined *a priori* as high risk: Bennett-Guerrerro *et al*^8^ only included high risk patients, defined as having diabetes and/or a body mass index (BMI) over 30kg/m^2^, while Friberg *et al^7^* included a subgroup defined as high risk as having diabetes and/ or a BMI over 25kg/m^2^.

### Any SWI in all participants

**Figure 1:**
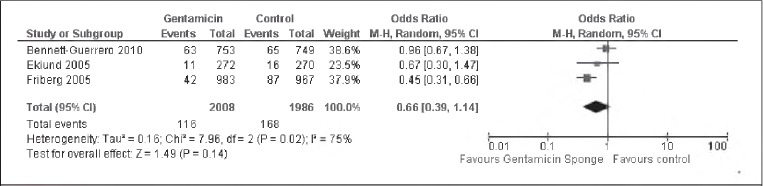
Pooled data analysis assessing incidence of all post-operative sternal wound infections in all participants. A random effects model was used because there was evidence of significant heterogeneity.

All three studies included outcome data on any SWI during the follow-up period. There was significant heterogeneity between the studies (I^2^=75%). Results from the random effects model are shown in Figure 1. There was some evidence of a difference between the intervention and control groups in the incidence of any post-operative SWI but this was not statistically significant (OR: 0.66, 95% Cl: 0.39–1.14).

### Deep SWIs in all participants

**Figure 2:**
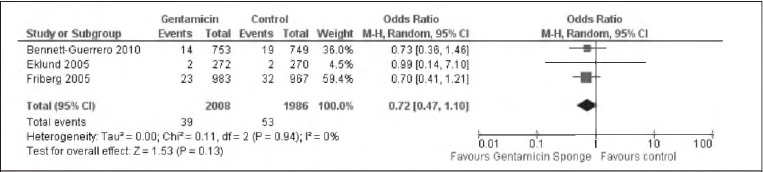
Pooled data analysis assessing incidence of deep post-operative sternal wound infections in all participants. The random effects model was identical to the fixed effects model.

Each of the three trials included outcome data on deep SWIs during the follow-up period. There was no evidence of het erogeneity between the studies (I^2^=0%). There was some evidence of a difference between the intervention and control groups in the incidence of deep SWIs but this was not statistically significant (OR: 0.72, 95% Cl: 0.47–1.10) (Fig 2).

### Any SWI in high risk participants

**Figure 3:**
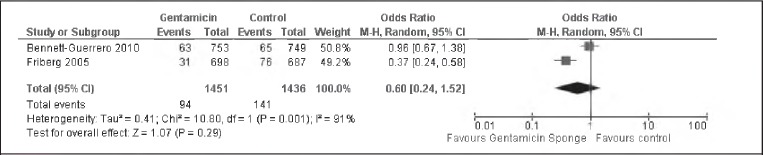
Pooled data analysis assessing incidence of all post-operative sternal wound infections in high risk participants. A random effects model was used because there was evidence of significant heterogeneity.

Two studies had participants who were considered *a priori*as high risk.^7^^–^^8^ There was evidence of significant heterogeneity between the two studies (I^2^=91%). From the random effects model, there was insufficient evidence to support a significant difference between the two groups in terms of any post-operative SWIs (OR: 0.60, 95% Cl: 0.24–1.52) (Fig 3).

### Deep SWIs in high risk participants

**Figure 4:**
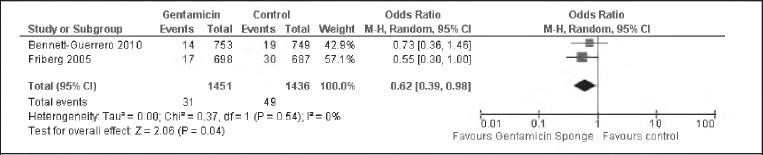
Pooled data analysis assessing incidence of deep post-operative sternal wound infections in high risk participants. The random effects model was identical to the fixed effects model.

The two studies with participants who were considered high risk also reported the incidence of deep SWIs.^7^^–^^8^ There was no evidence of heterogeneity between the studies (I^2^=0%). There was evidence of a statistically significant difference between the intervention and control groups in the incidence of deep post-operative SWI (OR: 0.62, 95% Cl: 0.390.98) (Fig 4).

## Discussion

Three RCTs (published between 2005 and 2010) were identified in this meta-analysis. None were from the UR and none were funded publicly. Data were pooled from 3,994 participants. When all participants were considered, there was some evidence that participants receiving a gentamicin impregnated collagen sponge (as well as prophylactic antibiotics) at the end of cardiac surgery had a lower incidence rate of any SWI but the difference was not statistically significant. There was, however, evidence of a significantly lower incidence of deep SWIs in high risk participants who received a gentamicin impregnated sponge.

There are a number of limitations to this review. The first is that only three studies met the criteria for inclusion. This precluded funnel plot analysis, which might have indicated publication bias.

The primary outcome for the studies (SWIs) was apparently defined similarly in the three included studies although the definitions involved subjective judgements from the assessors of the wounds. There were some differences between the trials in terms of their patient populations and interventions. One trial^8^ included only high risk patients and there was inter-trial variation in the baseline infection rates: compared with the UR, the incidence of infection in the control groups may be considered to be somewhat high.

The method of using the sponges also differed between studies. Two of the trials used two strips of collagen^7^^–^^8^ while the other study used only one (each containing 150mg gentamicin).^6^ Importantly, it appears that the sponges in the most recently published trial^8^ were softened in saline prior to implantation,^10^ probably altering the antibiotic concentration prior to and following implantation and therefore also the potential effectiveness. Furthermore, it is difficult to assess the effect of the sponge in isolation as there was apparently little standardisation of practice in relation to stabilisation of the sternum in individual studies, let alone across studies. It is well established that failure to achieve stabilisation of the sternum is associated with an increased incidence of post-operative infection.^11^

If gentamicin impregnated sponges were to be shown to be effective in reducing the incidence of SWIs, it would be important to investigate their cost effectiveness. Put simply, the upfront cost of the sponge would need to be offset against the costs associated with treating SWIs.

Only one study^7^ had an associated economic analysis.^12^ The most recently published study^8^ had planned a cost analysis but it was not undertaken given the study’s negative effectiveness results. Friberg *et al* concluded that despite the high cost of the gentamicin impregnated sponges, the use of two sponges, in addition to intravenous antibiotic prophylaxis, was cost effective, resulting in both lower costs and fewer infections for all patients as well as for high risk patients.^12^ However, this study was undertaken between 2000 and 2002 in Sweden and there may be changes in the relative costs as well as other potential variations in translating the findings to a UR setting. If it were definitively shown that the use of the sponges was clinically effective, it would be imperative to investigate their cost effectiveness in the UR setting.

In the current financial environment, it is likely that demonstration of cost effectiveness together with clinical effectiveness wouldprove the most potent driver for change in clinical practice. The potential implications are enormous given that over 182,000 cardiac surgical operations took place in the UR and Ireland between 2004 and 2009.^1^

## Conclusions

There is some evidence that the insertion of a gentamicin impregnated collagen sponge prior to closure of the ster num at the end of cardiac surgery, in addition to systemic antibiotics, reduces the incidence of any SWI and, in particular, there is evidence of a statistically significant reduction in the incidence of deep SWIs in high risk patients. However, there have been no publicly funded studies to date and there is still no published evidence of the clinical effectiveness or cost effectiveness in a UR setting. Further large, high quality, multicentre trials assessing both the clinical effectiveness and cost effectiveness of gentamicin impregnated sponges in reducing SWIs are required.

## Erratum

Hassan S, Wall A, Ayyaswamy B, Rogers S, Mills SP, Charalambous CP. Is there a need for early post-operative x-rays in primary total knee replacements? Experience of a centre in the UK. *Ann R Coll Surg Engl* 2011; **94**: 199–200.

In the above article published in the April issue of the Annals we erroneously printed the third author’s name as ‘Ayyawamy’ instead of ‘Ayyaswamy’. We apologise for the error and thank Brijesh Ayyaswamy for bringing this to our attention.
